# Crystal structures of Uso1 membrane tether reveal an alternative conformation in the globular head domain

**DOI:** 10.1038/s41598-020-66480-1

**Published:** 2020-06-12

**Authors:** Yoonyoung Heo, Hye-Jin Yoon, Hanseo Ko, Soonmin Jang, Hyung Ho Lee

**Affiliations:** 1 Department of Chemistry, College of Natural Sciences, Seoul National University, Seoul, 08826 Korea; 2 Department of Chemistry, Sejong University, Seoul, 05006 Korea

**Keywords:** Biochemistry, Structural biology

## Abstract

Membrane tethers play a critical role in organizing the complex molecular architecture of eukaryotic cells. Uso1 (yeast homolog of human p115) is essential for tethering in vesicle transport from ER to Golgi and interacts with Ypt1 GTPase. The N-terminal globular head domain of Uso1 is responsible for Ypt1 binding; however, the mechanism of tethering between ER transport vesicles and Golgi is unknown. Here, we determined two crystal structures for the Uso1 N-terminal head domain in two alternative conformations. The head domain of Uso1 exists as a monomer, as confirmed using size-exclusion chromatography coupled to multi-angle light scattering and analytical gel filtration. Although Uso1 consists of a right-handed α-solenoid, like that in mammalian homologs, the overall conformations of both Uso1 structures were not similar to previously known p115 structures, suggesting that it adopts alternative conformations. We found that the N- and C-terminal regions of the Uso1 head domain are connected by a long flexible linker, which may mediate conformational changes. To analyse the role of the alternative conformations of Uso1, we performed molecular docking of Uso1 with Ypt1, followed by a structural comparison. Taken together, we hypothesize that the alternative conformations of Uso1 regulate the precise docking of vesicles to Golgi.

## Introduction

Vesicular transport in eukaryotic cells is essential for communication between cellular organelles and contributes in many signalling pathways, which are mainly mediated by carrier vesicles coated by three types of proteins; coat-protein complex I (COP I), COP II, or clathrin-coated vesicles^1^. Diverse sets of cargo such as proteins and lipids are translocated to the endoplasmic reticulum (ER), and subsequently transported to their destinations via vesicular trafficking^2^. In the ER, the cargoes undergo folding and various modifications before being transported to the Golgi, where they are further modified. Therefore, transport from the ER to Golgi is an important step in vesicular transport for the maturation and correct trafficking of cargo^3^.

Cargo transport from the ER to Golgi is mediated by the COP II-coated vesicles^4^. The COP II coat complex consists of an inner shell (Sec23/Sec24) that sorts cargo into ER-derived vesicles and an outer cage (Sec13/Sec31) that leads to coat polymerization^5^. Once the vesicle is released from the ER, it should be delivered to and fuse with the correct target membrane. Budded vesicles are not transported to the target membrane randomly but in a directional manner, which is strictly regulated^6,7^. The Ypt/Rab GTPase-effector dependent system is one of the most well-characterized regulatory mechanisms^8^. Ypt/Rab GTPases mediate tethering of transport vesicles to target membranes in a nucleotide (GTP/GDP) bound manner^9^. GTP/GDP exchange causes a dramatic change in the conformation of switch 1 and switch 2 regions of the GTPases, enabling only GTP-bound GTPases to bind specific sets of effector proteins^10^. In yeast, the Rab1 homolog, Ypt1 interacts with its effector protein Uso1 and regulates COP II-coated vesicle trafficking^11^.

Uso1, a homolog of p115, is essential for tethering vesicle transport from ER to Golgi in yeast^12^. It was first isolated from temperature-sensitive secretory mutants in *yeast*, which showed a phenotype of blocked protein transport from ER to Golgi^13,14^. Requirement of Uso1 in vesicular transport was further demonstrated *in vitro* using semi-intact cells^11^. Interestingly, Uso1 was reported to participate not only in the tethering of ER-derived vesicles, but also in the sorting of glycosylphosphatidylinositol-anchored proteins at the exit from ER *in vitro*^15^. Uso1 is composed of 1,790 amino acids, which form a dimer with an N-terminal globular head, followed by a parallel coiled-coil^16^. The length of the coiled-coil region is approximately 154 nm, which might be long enough to tether the vesicles far from the Golgi^16^. Previous structural studies on the globular head domain of p115 revealed that it exists as a homodimer and exhibits an armadillo-fold that is decorated by elongated loops^17,18^. Dimeric p115 can interact with Rab1 in its GTP-bound state via its highly conserved HR1 domain and be recruited to COP II coated vesicles^17^.

The coiled-coil region of Uso1 contains two hinge regions (between 800–900 and 1,200–1,400) that can break the coiled-coil^16^. It is interesting to note that the collapse of the rod shape of EEA1, a coiled-coil tether, is regulated by the binding of the Rab5-GTP complex, where the hinge region is essential for correct vesicle trafficking from ER to Golgi^19^. The regulated bending of coiled-coils serves to increase the fidelity of vesicle trafficking^20^. Analogous to this, Uso1 might bend in its defined hinge regions by binding GTP-bound Ypt1. Consistent with this hypothesis, Uso1 has been reported to act as an inhibitor of fusion with target membranes when it is recruited to the wrong vesicles, and not by normal GTP-bound Ypt1^16,19^. However, it is unclear how Uso1 bending is regulated at the molecular level.

To understand how Uso1 interacts with Ypt1-GTP, we performed structural analysis of the N-terminal globular head region of Uso1 (Uso1^GHR^). Here, we present two crystal structures of Uso1^GHR^, which adopt alternate conformations distinct from previously reported structures of p115. Biochemical studies including size-exclusion chromatography coupled to multi-angle light scattering (SEC-MALS), analytical gel filtration, and molecular docking showed that the alternative conformations of Uso1^GHR^ are inappropriate for dimerization and should adapt to its partner via its degree of flexibility, providing insights into the regulation of Uso1 for precise vesicle tethering from ER to Golgi.

## Results and discussion

### **Structure determination of two different forms of Uso1**^**GHR**^**from*yeast***

**Figure 1:**
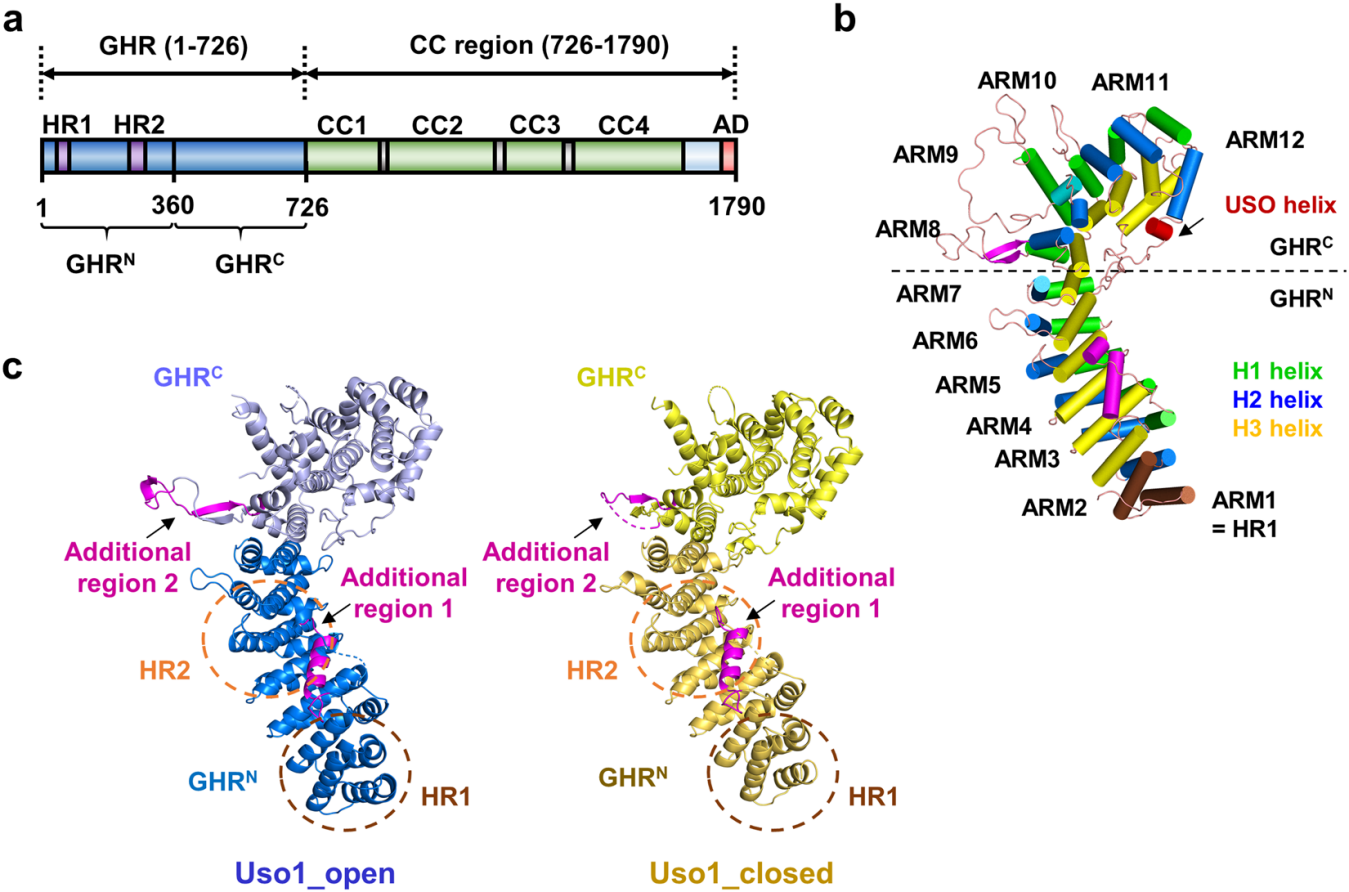
Overall structure of yeast Uso1^GHR^. (**a**) A schematic diagram of the full length Uso1 protein. GHR, CC, and AD denote globular head region, coiled-coil region, and acidic domain, respectively. HR1 and HR2 are highly conserved regions in Uso1 and p115 proteins. GHR^N^ and GHR^C^ are the N- and C-terminal regions (residues 1–360 and 360–726) of GHR domain, respectively. The construct used for structure determination is shown in blue. (**b**) Overall structure of yeast Uso1^GHR^ (Uso1_open), consisting of 12 armadillo (ARM) repeats. Each ARM repeat consists of 3 helices; H1 (green), H2 (blue), and H3 (yellow). HR1 and USO1 helix are denoted in brown and red, respectively. Additional regions 1 and 2 are shown in magenta. (**c**) Ribbon diagrams of Uso1^GHR^ (Uso1_open and Uso1_closed). GHR^N^ and GHR^C^ are denoted by different colours. Additional regions 1 and 2 are shown in magenta. The positions of HR1 and HR2 are indicated by dotted circles.

**Figure 2:**
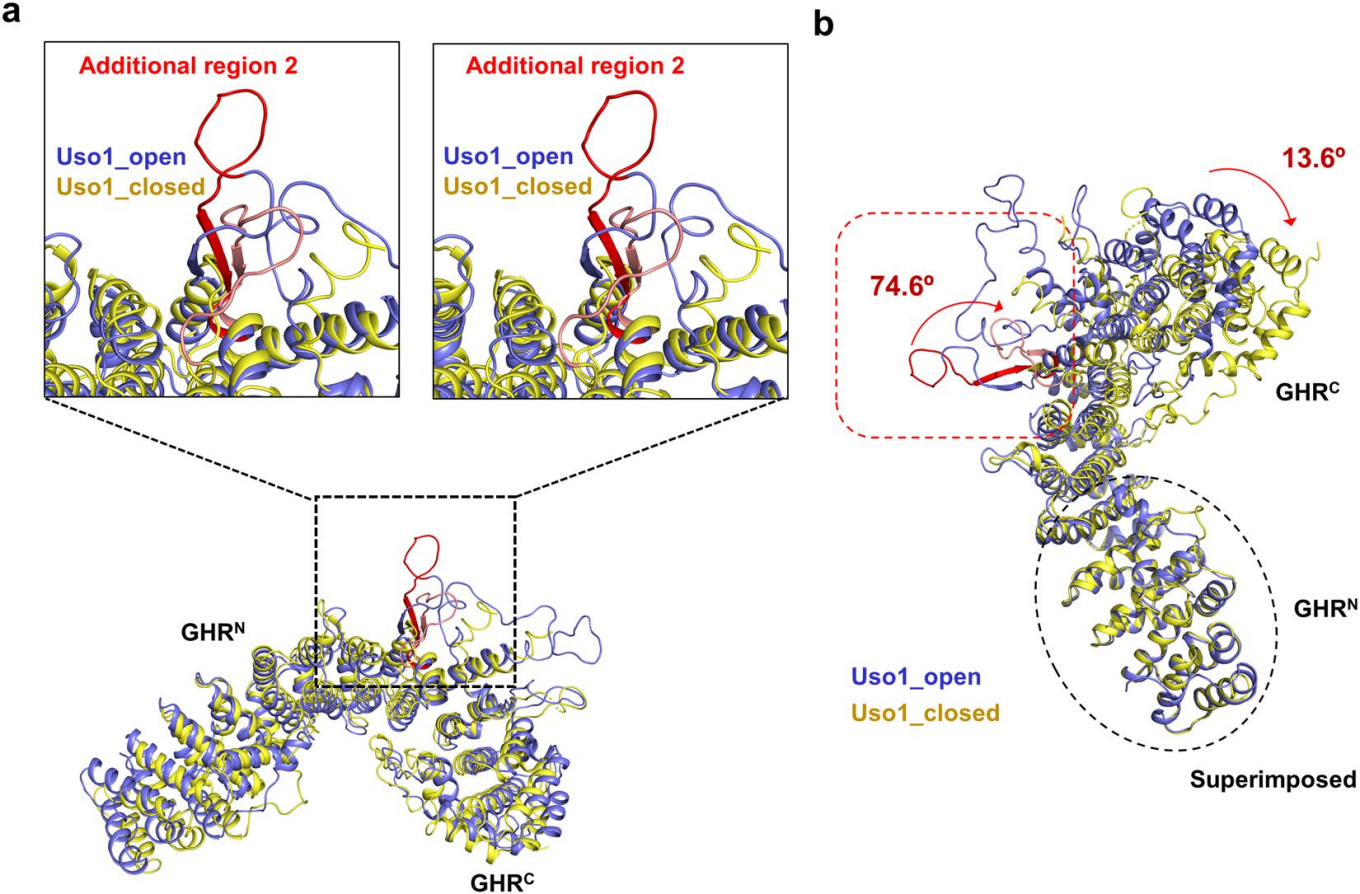
Structural comparison of two crystal structures of Uso1^GHR^ (Uso1_open and Uso1_closed). (**a**) Magnified stereo view showing details in additional region 2. Overall Cα positions of Uso1_open and Uso1_closed are superimposed (light blue and yellow, respectively). Additional region 2 is denoted in red. (**b**) Structural comparison of Uso1_open and Uso1_closed. The N-domains are superimposed, and the rotation angle is indicated in the C-domain. The superposition of Uso1_closed (yellow) to Uso1_open (light blue) reveals an inter-domain rotation of approximately 13.6°, compared to Uso1_open.

Two different crystal structures of the globular head domain of Uso1 (Uso1^GHR^, Fig. 1a) were determined by the molecular replacement method, using the globular head region of human p115 (PDB ID: 2W3C) as probe^18^. Both Uso1^GHR^ models exist as monomers in an asymmetric unit (Fig. 1). Overall, the Uso1^GHR^ structures were revealed to have a right-handed, α-solenoid structure; however, they adopted two different conformations—open and closed forms (hereafter referred to as Uso1_open and Uso1_closed, respectively). The open and closed forms were defined according to the conformational difference in the C-terminal domain of Uso1^GHR^ (GHR^C^) when each N-terminal domain of Uso1^GHR^ was superimposed. To see how open and closed forms are quantitively different each other, we analysed the inter-domain rotation of GHR^C^ in Uso1^GHR^ using DynDom program^21^, which analyses conformational changes in proteins containing hinge-bending regions. In this analysis, the fixed domains were defined as residues 17–363 (GHR^N^) and the moving domains as residues 364–715 (GHR^C^). Thereafter, we analysed the relative positions of GHR^C^ domains compared to the superimposed GHR^N^. GHR^C^ of Uso1_closed showed more clockwise rotation (approximately 13.6°) than that of Uso1_open (Figs. 1c and 2b). According to the rotation degree of GHR^C^ based on GHR^N^, we defined the structure with less rotated GHR^C^ as Uso1_open and that with more rotated GHR^C^ as Uso1_closed.

For Uso1_open, residues 1–4 of the N-terminus; 120–122, 459–489, and 546–550 of the flexible loop; and 720–726 of the C-terminus cannot be seen in the structure, suggesting that they are disordered (Fig. 1c). For Uso1_closed, residues 1–14 of the N-terminus, 116–123, 385–391, 456–490, and 544–550 of the flexible loop, and 719–726 of the C-terminus were not seen in the structure (Fig. 1c). Because of weak electron density in the loops in the middle of GHR^N^ and GHR^C^, we decided not to include them in our final model for Uso1_open (PDB ID 6LSU). However, these loops have not been resolved in other Uso1 homologs, so we refined another model for Uso1_open, which contained all residues except the N- and C-terminal parts, for analysis and designated it as Uso1_open (2) in Table S1 (Fig. 2).

### Overall structure of Uso1^GHR^

Upon analysing the overall structures of both Uso1^GHR^ models (Uso1_open and Uso1_closed), we found that Uso1^GHR^ consisted of multiple α-helices and loops forming a β-catenin-like armadillo-fold (Fig. 1b)^18^. The super-helical α-solenoid of Uso1^GHR^ is structurally related to β-catenin and karyopherin-α/importin-α proteins^22–24^. To further characterize the structural and functional features of Uso1^GHR^, we performed a structural similarity search for Uso1^GHR^ using the DALI server^25^, which has been used to identify several armadillo-fold proteins, including bovine p115 (PDB code 3GQ2)^17^, importin (PDB code 4B8J)^26^, and plakophilin (PDB code 2I99)^27^. Among them, bovine p115 showed the highest Z-score (30.9).

The overall shape of Uso1^GHR^ is quite similar to other structurally characterized mammalian p115 proteins^17,18^, and we observed 12 armadillo repeats (ARM1–ARM12). Each armadillo repeat was composed of three right-handed α-helices (H1–H3) except for ARM1 and ARM2, and several discrepancies were noted, when compared to p115 (Fig. 1b). Interestingly, two specific regions (extending from residues 92–113 and 384–400) had largely distinct conformations from mammalian p115 proteins. One of them was an additional α-helix (coloured in purple), distinct from the classical armadillo repeat and absent in mammalian homologs, was present between H3 of ARM2 and H1 of ARM3 (Fig. 1c). The other region (residues 384–400), between H1 and H2 in ARM8, had a longer loop than that of p115. This region is structurally disordered in p115.

Uso1^GHR^ can be separated into two parts; the N-terminal region (ARM1–ARM7 and GHR^N^) and the C-terminal region (ARM8–ARM12 and GHR^C^) based on its structural similarity with other armadillo-fold proteins (Fig. 1b). The N-terminal region has two highly conserved homologous regions (HR1 and HR2) (Fig. 1c and Supplementary Fig. 1). HR1 (residues 18–51) interacts with GTPase Ypt1^11^, and HR2 (residues 219–271) recognizes the COG complex, COG2^28^. The GHR^C^ contains longer loops between H2 and H3 than those of GHR^N^, which induces a curvature in the C-terminal region, resulting in an armadillo-like shape (Fig. 1 and Supplementary Fig. 1). ARM12 consisted of 4 helices, unlike other repeats, which contain 3 helices, and does not follow the rule of classical armadillo repeat proteins. Among the 4 helices, the USO helix resides on the interface of the Uso1 homodimer (Fig. 1b), which was shown to be crucial for homodimer formation^18^.

### Structural comparison of two crystal structures with distinct conformations

**Figure 3:**
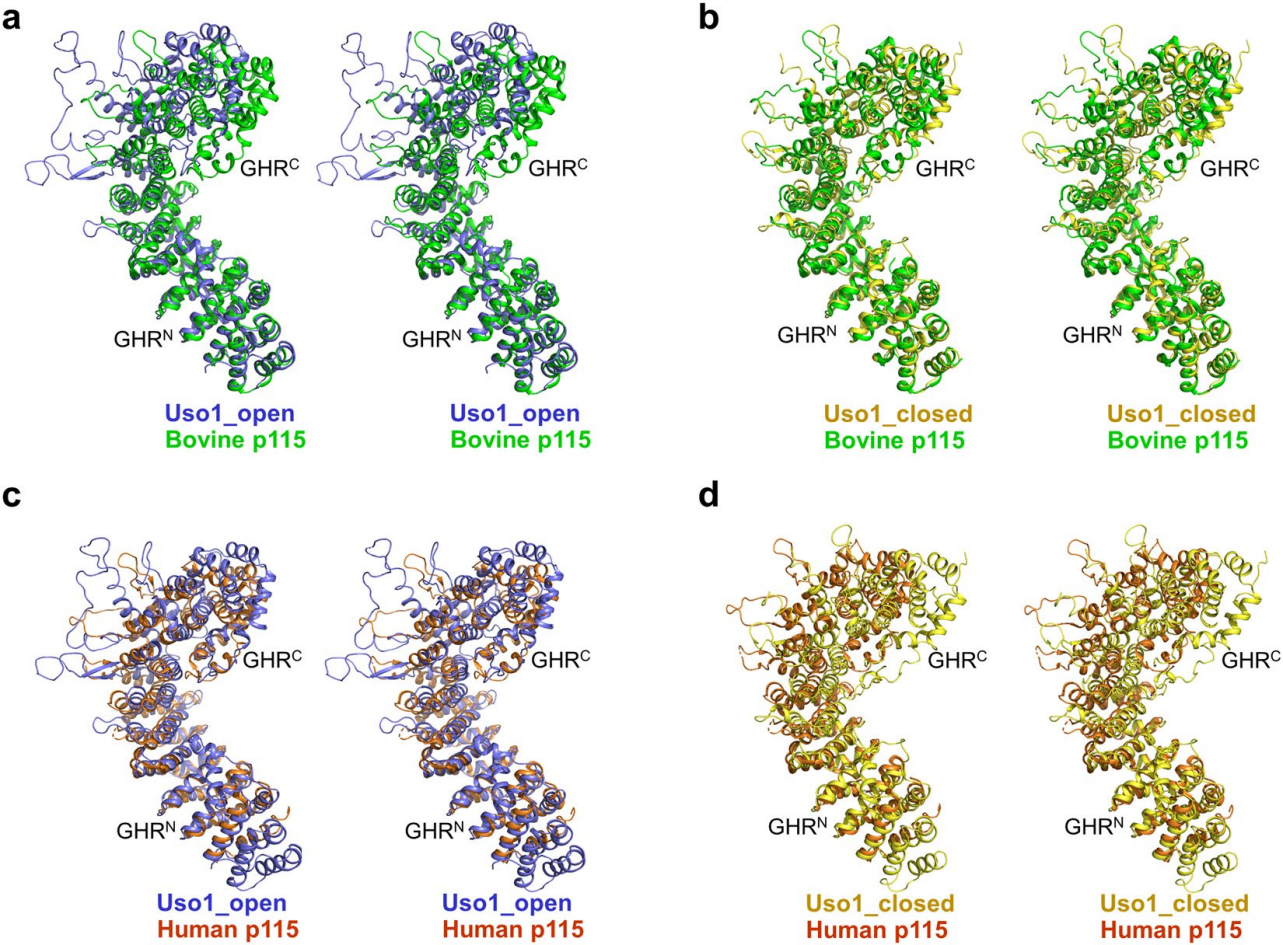
Structural comparisons of Uso1^GHR^ with p115^GHR^. (**a**) Structural comparison of Uso1_open (light blue) and bovine p115^GHR^ (PDB code 3GQ2, green). All figures are drawn in stereo. (**b**) Structural comparison of Uso1_closed (yellow) and bovine p115^GHR^ (PDB code 3GQ2, green). (**c**) Structural comparison of Uso1_open (light blue) and human p115^GHR^ (PDB ID: 2W3C, orange). (**d**) Structural comparison of Uso1_closed (yellow) and human p115^GHR^ (PDB ID: 2W3C, orange).

When both structures of Uso1^GHR^ were compared by superimposing their overall Cα positions, overall structures look similar, with an rmsd of 3.10 Ǻ for 636, equivalent Cα positions (Fig. 2a). However, when we superimposed GHR^N^ domains of Uso1_open and Uso1_closed (rmsd 0.62 Ǻ for 312 equivalent Cα atoms), the overall conformations of GHR^C^ domains were significantly different from each other (Fig. 2b). When both structures of Uso1^GHR^ were compared with those of human (PDB code 2W3C) and bovine (PDB code 3GQ2) p115 by superimposing overall Cα positions (Uso1_open/human p115: rmsd 3.88 Ǻ for 447 equivalent Cα atoms, Uso1_closed/human p115: rmsd 3.43 Ǻ for 436 equivalent Cα atoms, Uso1_open/bovine p115: rmsd 4.66 Ǻ for 521 equivalent Cα atoms, Uso1_closed/bovine p115: rmsd 3.11 Ǻ for 480 equivalent Cα atoms), there were only small differences between the two Uso1^GHR^ structures and those of mammalian p115 proteins, in the same way between Uso1_open and Uso1_closed. However, when we compared them by superimposing their GHR^N^ domains, the structure of Uso1_open was more similar to human p115 (PDB entry 3GQ2) than that of Uso1_closed (Fig. 3a,c); the structure of Uso1_closed was more similar to bovine p115 (PDB entry 2W3C) than that of Uso1_open (Fig. 3b,d). Interestingly, however, we observed that the relative positions of GHR^N^ of Uso1^GHR^ (Uso1_open and Uso1_closed) were distinct from those of previously reported structures of mammalian p115 proteins^17,18^. Taken together, we concluded that the inter-domain rotation between GHR^N^ and GHR^C^ generates diverse conformations of GHR domain.

To understand the structural features responsible for different conformations of GHR, we analysed the inter-domain rotations using the DynDom program^21^. We observed that the conformations of the specific loop (residues 384-400) were significantly different in Uso1_open and Uso1_closed, as indicated by red and pink loops (74.6° clockwise rotation), respectively (Fig. 2b). We hypothesized that the additional region 2 of Uso1^GHR^, which is absent in p115^GHR^, might provide flexibility to Uso1^GHR^, inducing the two conformations of Uso1_open and Uso1_closed. However, this hypothesis needs to be validated by further studies.

### **Oligomeric state of Uso1**^**GHR**^**in solution**

Full-length Uso1 exists as a stable homodimer, with an N-terminal globular head region and a C-terminal coiled-coiled region, as seen in EM images^16^. Notably, electron microscopy studies have shown that Uso1 has a single or double globular domain^16^; we speculated that these corresponded with the monomeric and dimeric forms of Uso1^GHR^, respectively. Undoubtedly, the long C-terminal coiled coil is responsible for homo-dimerization of Uso1; however, it is uncertain whether Uso1^GHR^ exists as dimer in solution when it is expressed without the C-terminal coiled coil. Previous structural studies with bovine p115 report that it was purified as either a monomer or dimer in solution, and used for crystallization separately^17^. This suggests that the N-terminal head domain does not form a stable dimer, and there might be an interconversion between the monomer and dimer.

**Figure 4:**
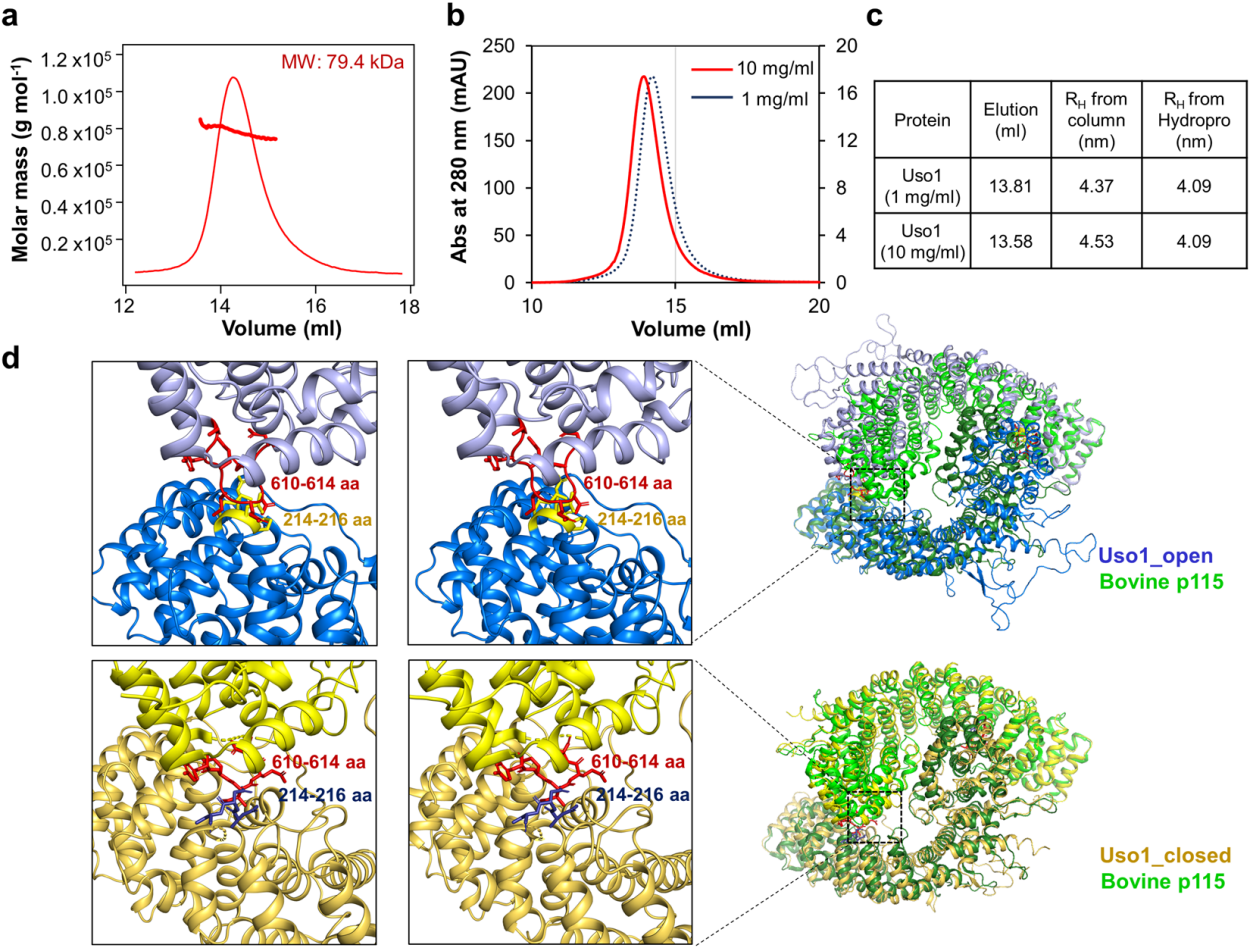
Oligomeric state of Uso1^GHR^ in solution. (**a**) The oligomeric state of Uso1^GHR^ in solution was analysed by SEC-MALS. The thick line represents measured molecular mass. The expected molecular mass theoretically is 84 kDa. (**b**) Analytical gel filtration profiles of Uso1^GHR^ at high (10 mg/mL; red solid line) and low (1 mg/mL; blue dotted line) concentrations. (**c**) R_H_ from HYDROPRO and Stokes radii calculated from structure models for Uso1^GHR^ in different concentrations. (**d**) Magnified stereo view showing clash in dimeric Uso1^GHR^ models. In the upper panel, two Uso1_open monomers (blue) have been superimposed onto each subunit of bovine p115 (PDB code 3GQ2, green). Clashed regions (residues 214–216 and 610–614) are magnified in the left panel and shown as red and yellow sticks. In the lower panel, two Uso1_closed monomers (yellow) have been superimposed onto each subunit of bovine p115 (PDB code 3GQ2, green). Clashed regions, residues 214–216 and 610–614, have been magnified in the left panel and shown as red and dark blue sticks, respectively.

To analyse the oligomeric state of Uso1^GHR^, we measured the molecular weight of Uso1^GHR^ using SEC-MALS (Fig. 4a). The measured molecular mass of Uso1^GHR^ was 79.4 kDa, close to the theoretical value of 84 kDa, which indicates that Uso1^GHR^ exists as a monomer in solution. We next hypothesized that the oligomeric state of Uso1^GHR^ in solution may depend on its concentration. To analyse the concentration dependent quaternary structure of Uso1^GHR^ in solution, analytical gel filtration was performed in high and low concentrations of Uso1^GHR^ (10 and 1 mg/mL, respectively) using a Superdex 200 (10/300 GL) column (Fig. 4b). The theoretical R_H_ value (hydrodynamic radius) of monomeric and dimeric Uso1^GHR^ were 4.09 nm and 5.31 nm, respectively, when calculated using the HYDROPRO program^29^. From analytical gel filtration chromatography, the experimental R_H_ value of Uso1^GHR^ was 4.37 nm in low concentration (1 mg/mL) and 4.53 nm in high concentration (10 mg/mL) (Fig. 4c). These experimental R_H_ values are close to the theoretical R_H_ value of the Uso1^GHR^ monomer. Thus, we concluded that Uso1^GHR^ existed as a monomer in concentration-independent manner. Consistently, Uso1^GHR^ is also monomer in an asymmetric unit of crystal regardless of its symmetry operation. Taken together, Uso1^GHR^ only exists as a monomer in solution and the dimeric form might be assembled by the help of the C-terminal coiled coil domain. When we made a dimeric model of Uso1^GHR^ by superimposing the structures of Uso1^GHR^ with that of dimeric bovine p115 (PDB code 3GQ2), a steric clash between residues 214–216 of one subunit and residues 610–614 of the other subunit was observed, suggesting that the current conformations of Uso1^GHR^ are inappropriate for dimerization and should adapt to its partner via its degree of flexibility (Fig. 4d).

### Integrative modelling of the Uso1 and Ypt1 interaction

We next investigated how Uso1^GHR^ makes a complex with Ypt1. As we failed to purify the Uso1^GHR^-Ypt1 complex for crystallization, we adopted the protein-protein docking approach to make a complex model of Uso1^GHR^ and Ypt1. Uso1_open (PDB code 6LSU) and GTP-bound Ypt1 (PDB code 1YZN)^30^ were used as the starting models. To increase accuracy of protein-protein docking, experimental data from immunoprecipitation studies, which identified the crucial residues of p115 for Rab1 binding^17^, were used for inter-molecular distance restraints. In case of Ypt1, GTP/GDP exchange causes a dramatic change in the conformation of switch 1 and switch 2 regions, enabling only GTP-bound GTPases to bind specific sets of effector proteins^10,31^. Switch 1 and switch 2 of Ypt1 regions have been reported to be crucial for binding the Rab GDP-dissociation inhibitor; thus, we reasoned that the highly flexible switches might also contribute to Uso1 binding. Based on these observations, we defined active sites for Uso1^GHR^ (Glu18, Arg26, Asp35, Arg36, Lys44, and Arg48) and Ypt1 (Asp44, Trp62, and Asp63). After several rounds of molecular docking using the HADDOCK program^32^, we could obtain four reliable docking models with low energy scores of approximately −130 kcal/mol.

**Figure 5:**
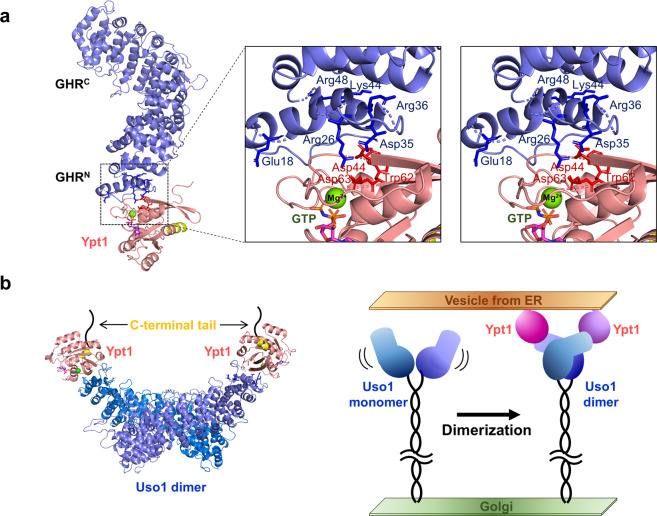
Integrative modelling of the Uso1 and Ypt1 interaction. (**a**) Overall docking model of Uso1^GHR^ (Uso1_open, light blue) and yeast Ypt1 (PDB entry 1YZN, salmon). Detailed interactions have been magnified and viewed in stereo. The active residues for Uso1^GHR^ and Ypt1 are shown as blue and red sticks, respectively. Mg^2+^ ion and GTP are shown as a green ball and stick. (**b**) The composite model of the Uso1-Ypt1 complex in the ER-Golgi vesicle tethering pathway. In the left panel, dimer model of Uso1^GHR^ is generated by manually fitting the Uso1^GHR^-Ypt1 complex model into bovine p115^GHR^ (PDB code 3GQ2). Overall model of the Uso1-Ypt1 complex between the Golgi and vesicles from ER is shown in the right panel.

Among the four candidates, we selected the final docking model by considering two criteria; first, the C-terminal region of Ypt1, where the C-terminal prenyl lipid is linked for membrane anchoring, should face the membrane of the ER-derived vesicle. Second, the active residues should reside at the interface of Uso1 and Ypt1. To select the most reasonable model, four candidates were superimposed onto the dimer model of p115 (PDB code 3GQ2) and the direction of the C-terminal region in Ypt1 was checked. Consequently, a reliable docking model with a low energy score (−132 kcal/mol), that satisfied the two criteria was selected (Fig. 5). When we examined the docking model of the Uso1^GHR^-Ypt1 complex, the protruding loops of Ypt1, consisting of switch 1 and switch 2, were reasonably docked to the HR1 surface of Uso1^GHR^ and C-terminus of Ypt1 was surface-exposed so that it could be further linked to the vesicular membrane from the ER (Fig. 5a). We also observed the interactions of active residues (Arg26/Asp35 of Uso1 and Asp44/Asp63 of Ypt1) (Fig. 5a). However, it should be noted that we might have a different docking model if either of the two criteria is incorrect.

Based on the docking model and experimental data, we suggested an integrative model of Uso1 and Ypt1 interaction (Fig. 5b). Without Ypt1, Uso1^GHR^ could exist as a flexible monomer, which changes its conformations alternatively. When vesicles from the ER are delivered to Uso1, Uso1^GHR^ can form a dimer by interacting with Ypt1, which might induce docking and fusion. Although additional biochemical and structural studies are needed to fully understand the regulation mechanism of Uso1, our data provide a foundation for further investigation.

## Methods

### Cloning and protein preparation

Uso1 gene (amino acids 1–726) was amplified from the genomic DNA of *Saccharomyces cerevisiae* by polymerase chain reaction and cloned into the pGST2 expression vector. This construct codes for an N-terminal GST tag and the Uso1 gene under the control of T7 promoter. The Uso1 protein was expressed in *Escherichia coli* strain BL21(DE3) cells induced with 1 mM isopropyl-β-D-1-thiogalactopyranoside and expressed at 18 °C for 16 h. Cells were lysed by passing them through a microfluidizer in buffer A (20 mM Tris-HCl (pH 8.0) and 200 mM NaCl) containing 1 mM PMSF. The lysed cells were centrifuged at 4,611 ×*g* (Vision V506CA rotor) for 30 min at 277 K to pellet cell debris; the supernatant was applied to a GST affinity resin (GE Healthcare). Proteins were eluted with buffer A, containing 15 mM GSH solution, and the TEV protease was used to treat eluted protein for 18 h at 277 K to remove the GST tag. To separate the cleaved GST tag from Uso1, a further purification step was done using HiTrap Q HP column (5 mL; GE Healthcare), which was previously equilibrated with 20 mM Tris-HCl (pH 8.0) and 50 mM NaCl. GST-tag cleaved Uso1 protein was eluted with a linear gradient of 50~500 mM NaCl (10 column volumes). Further purification was performed by size exclusion chromatography (HiLoad 16/600 Superdex 200 prep grade, GE Healthcare), which was previously equilibrated with buffer A containing 5 mM β-mercaptoethanol. Peak fractions containing the Uso1 protein were pooled and concentrated to 20 mg/mL for crystallization. The extinction coefficient of Uso1 was calculated with ProtParam (http://web.expasy.org/protparam/).

### Crystallization, structure determination, and refinement

Crystals of Uso1_open protein (open form) were grown at 298 K using the sitting drop method, by mixing 0.45 µL of Uso1 protein (20 mg/mL) with 0.45 µL of reservoir solution, consisting of 0.2 M bis-Tris HCl (pH 6.0) and 2 M sodium formate with 0.1 µL of sodium fluoride as an additive. The crystals were transferred to a solution containing the reservoir solution and 15% sucrose for cryoprotection. Data were collected at 100 K in 1° oscillations at the 7A beamline of the Pohang Light Source. Uso1_open protein crystal was diffracted to a resolution of 2.7 Å. The diffraction data were processed and scaled using the HKL2000 software package^33^. The crystal for Uso1_open belonged to space group *P*3_1_21, with unit cell parameters of *a* = 104.4 Å, *b* = 104.4 Å, and *c* = 231.8 Å. The structure was solved using the molecular replacement method using human p115 model (PDB ID: 2W3C) as probe^34^. Subsequent manual model building was performed using the COOT program^35^ and restrained refinement was carried out using the PHENIX program^36^. Several rounds of model building, simulated annealing, positional refinement, and individual B-factor refinement were performed using the COOT and PHENIX programs. The atomic coordinates and structure factors were deposited in the Protein Data Bank (PDB code 6LSU).

Crystals of Uso1_closed (closed form) were grown at 298 K using the hanging drop method, by mixing 1 µL of Uso1 protein (20 mg/mL) with 1 µL of reservoir solution consisting of 0.1 M Tris-HCl (pH 7.0), 0.2 M MgCl_2_, and 2.5 M NaCl. The crystals were transferred to a solution containing the reservoir solution and 10% glycerol for cryoprotection. Data were collected at 100 K in 1° oscillations at the 7A beamline of the Pohang Light Source. Uso1_closed crystal was diffracted to a resolution of 2.94 Å. The diffraction data were processed and scaled using the HKL2000 software package^33^. The crystal for Uso1_closed belonged to the space group *P*3_1_21, with unit cell parameters of *a* = 114.4 Å, *b* = 114.4 Å, and *c* = 193.4 Å. The methods for structure calculation were identical with those used for Uso1_open and the atomic coordinates and structure factors were deposited in the Protein Data Bank (PDB code 6LST). Table S1 lists the refinement statistics. Atomic coordinates and structure factors for Uso1_open and Uso1_closed proteins have been deposited in the Protein Data Bank (PDB ID codes 6LSU and 6LST, respectively).

### Size exclusion chromatography with multi-angle light scattering (SEC-MALS)

SEC-MALS experiments for Uso1 were performed using an FPLC system (GE Healthcare) connected to a Wyatt MiniDAWN TREOS MALS instrument and a Wyatt Optilab rEX differential refractometer. A Superdex 200 10/300 GL (GE Healthcare) gel-filtration column was pre-equilibrated with buffer A containing 5 mM β-mercaptoethanol and normalized using ovalbumin. Proteins (1 mg) were injected at a flow rate of 0.4 mL/min. Data were analysed using the Zimm model for static light-scattering data fitted and graphed, using EASI graph with a UV peak, in the ASTRA V software (Wyatt).

### DynDom analysis

The domain movement of Uso1_open and Uso1_closed structures were analyzed using the program, DynDom. Three main parameters were varied in this program: the window length (9 residues), the minimum ratio (1.0), and the minimum domain size (20 residues). With these default values, the fixed domains were defined as residues 17–363 (GHR^N^) and the moving domains as residues 364–715 (GHR^C^).

### Analytical gel filtration

Purified Uso1 protein was subjected to analytical gel filtration chromatography on a Superdex 200 (10/300 GL) column, pre-equilibrated with buffer A with 5 mM β-mercaptoethanol, at a constant flow rate of 0.5 mL/min. The standard curve was obtained using molecular weight markers (Sigma). Stokes radii of β-amylase, alcohol dehydrogenase, carbonic anhydrase, and cytochrome c were calculated from the crystal structures of each protein (PDB codes: 1FA2, 2HCY, 1V9E, and 1HRC, respectively) by using the HYDROPRO program^29^.

### Sequence alignments

The sequence of *S. cerevisiae* Uso1 was compared to other mammalian homologs of p115. The UniProtKB/Swiss-Prot accession numbers of the sequences used are P25386 (yeast), P41541 (bovine), O60763 (human), Q9Z1Z0 (mouse), and P41542 (rat). Sequences were aligned using ClustalW^37^ and secondary structure elements were assigned by PyMOL (The PyMOL Molecular Graphics System, http://www.pymol.org).

### Molecular docking

HADDOCK version 2.2^32^ was used for protein-protein docking studies. HADDOCK was also used to refine the docked structures, starting from the randomly generated initial structures. This process included rigid body docking, followed by semi-flexible searching using simulated annealing, especially at the interface region. The docking process was completed by consideration of water solvation. During this process, the number of possible docked structures was narrowed down based on the docking scores. The default number of initial structures generated in rigid body docking was 2,000 and the best 400 structures were subjected to semi-flexible docking. Finally, 200 possible docked structures were obtained for analysis after consideration of water solvation. For better results, we have changed these values to 2,000, 400, and 200, respectively. As for the parameters for HADDOCK, we have used the default 5.4 version of protein and solvent topologies as implemented in HADDOCK 2.2 throughout the docking procedure. Full length Uso1_open (PDB ID: 6LSU) and GTP-bound Ypt1 (PDB ID: 1YZN) were used as docking models^30^. We used active sites Glu18, Arg26, Asp35, Arg36, Lys44, and Arg48 for Uso1 and Asp44, Trp62, and Asp63 for Ypt1 with no passive sites. For the analysis of the docked structures, we used the fraction of common contact based cluster analysis^38^ as incorporated in HADDOCK. In fraction of common contact, the structural similarity for clustering was based on atomic contact with a pre-defined distance as contact threshold^38^. The structure figures were plotted using the program PyMOL (http://pymol.sourceforge.net).

## Supplementary information

Supplemental information.
