# Supplementary material for: Crystal structures of Uso1 membrane tether reveal an alternative conformation in the globular head domain
Source: Sci Rep. 2020 Jun 12;10:9544. doi: 10.1038/s41598-020-66480-1 (PMC7293329; doi:10.1038/s41598-020-66480-1)
Supplement: Supplementary file 1 — Supplemental information. [file 41598_2020_66480_MOESM1_ESM.docx]

**Crystal structures of Uso1 membrane tether reveal an alternative conformation in the globular head domain**

Yoonyoung Heo^1,*^, Hye-Jin Yoon^1,*^, Hanseo Ko^1^, Soonmin Jang^2^ and Hyung Ho Lee^1,†^

^1^Department of Chemistry, College of Natural Sciences, Seoul National University, Seoul 08826, Korea

^2^Department of Chemistry, Sejong University, Seoul 05006, Korea

^*^These authors contributed equally to this work.

^†^Corresponding author:

Professor Hyung Ho Lee, Department of Chemistry, College of Natural Sciences, Seoul National University, Seoul 08826, Korea

Correspondence to Hyungholee@snu.ac.kr

*Keywords*: Membrane tether, vesicle transport, Uso1, Ypt1

**Supplementary figures**

**
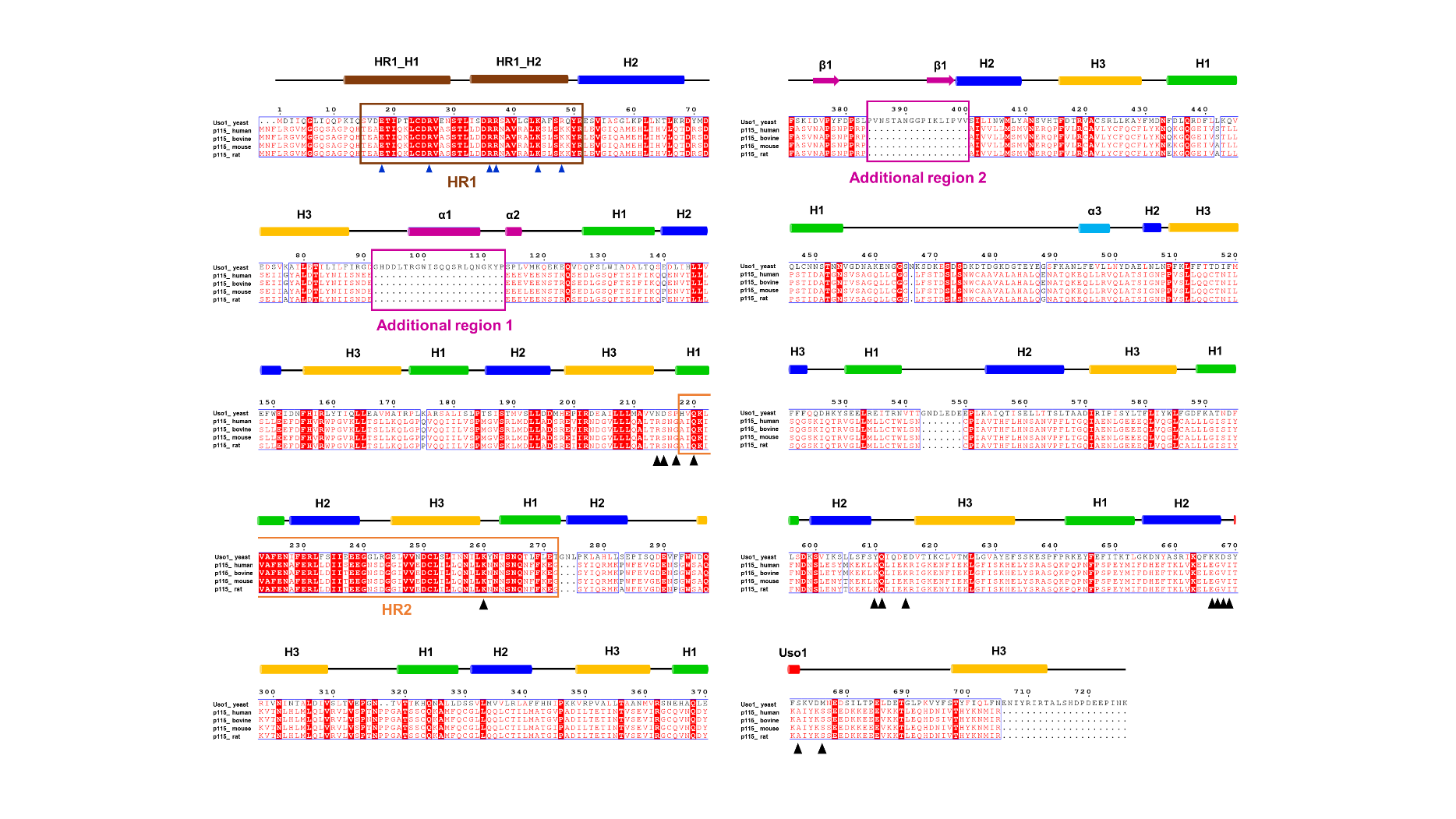
**

**Figure S1.** **Sequence alignment of Uso1^GHR^ with p115^GHR^ in mammalian species.** Multi-alignment of Uso1^GHR^ from *Saccharomyces cerevisiae* (UniProtKB/Swiss-Prot accession number P25386) against p115^GHR^ from *Homo sapiens* (UniProtKB/Swiss-Prot accession number O60763), *Bos taurus* (UniProtKB/Swiss-Prot accession number P41541), Mus musculus (UniProtKB/Swiss-Prot accession number Q9Z1Z0), and *Rattus norvegicus* (UniProtKB/Swiss-Prot accession number P41542). Highly (100%) conserved residues highlighted in red box with white color of characters and semi-conserved residues (80%) are highlighted with red color of characters. The boxes denoted by different colors represent sequences for HR1, HR2, and additional regions. The active residues used in molecular docking are represented by blue triangles below the sequences.


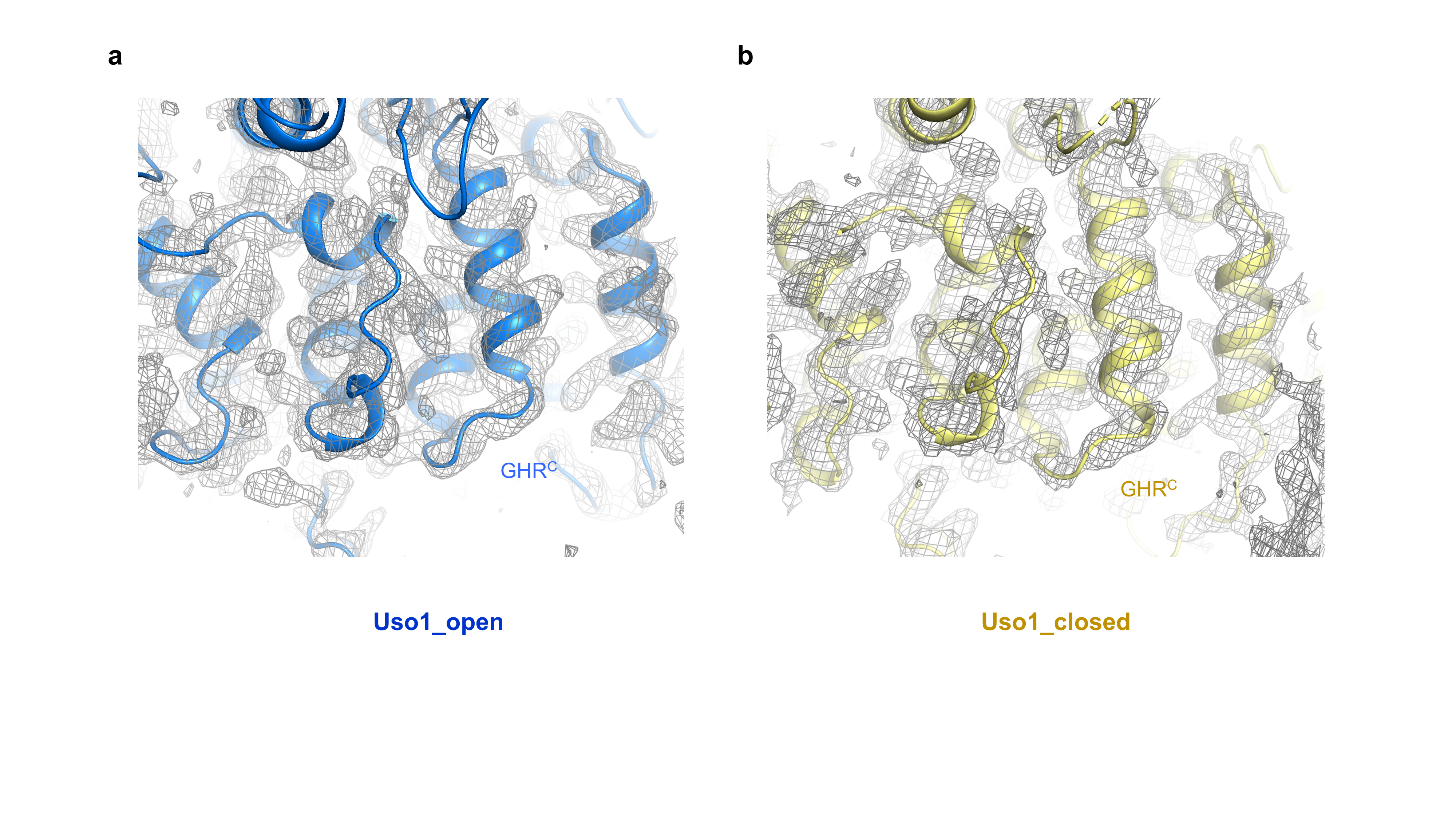


**Figure S2.** **Electron density map (2Fo-Fc, 1σ) for Uso1_open and Uso1_closed structures.** (**a**) Electron density map for GHR^C^ of Uso1_open (light blue). (**b**) Electron density map for GHR^C^ of Uso1_closed (yellow).

**Table S1. Statistics for data collection and refinement**

| Data set | Uso1_open (1) | Uso1_open (2) | Uso1_closed |
| --- | --- | --- | --- |
| ***A. Data collection*** |  |  |  |
| X-ray source | PLS 7A |  | PLS 7A |
| X-ray wavelength (Å) | 1.0000 |  | 1.0000 |
| Space group | *P*3_1_21 |  | *P*3_1_21 |
| Unit cell length (*a*, *b*, *c*, Å) | 104.4, 104.4, 231.8 |  | 114.4, 114.4, 193.4 |
| Unit cell angle (α, β, γ, °) | 90, 90, 120 |  | 90, 90, 120 |
| Resolution range (Å) | 50-2.70 |  | 50-2.94 |
| Total / unique reflections | 243,939/77,635 |  | 282,039/31,647 |
| Completeness (%) | 99.8 (100.0)^a^ |  | 98.7 (97.9)^b^ |
| Average *I*/σ (*I*) | 22.2 (2.0)^a^ |  | 42.3 (4.2)^b^ |
| *R*_merge_*^c^* (%) | 8.7 (82.9)^a^ |  | 8.8 (89.6)^b^ |
|  |  |  |  |
| ***B. Model refinement statistics*** |  |  |  |
| Resolution range (Å) | 50-2.7 | 50-2.7 | 50-2.94 |
| *R*_work_ / *R*_free_*^e^*(%) | 19.8 / 23.0 | 19.9/23.4 | 19.2 / 25.2 |
| Number / average *B*-factor (Å^2^) |  |  |  |
| Protein nonhydrogen atoms | 5,449/81.0 | 5,810/84.9 | 5,222/79.8 |
| Water oxygen atoms | 124/75.4 | 124/73.2 | 111/68.7 |
|  |  |  |  |
| R.m.s. deviations from ideal geometry |  |  |  |
| Bond lengths (Å) | 0.011 | 0.011 | 0.012 |
| Bond angles (°) | 1.742 | 1.732 | 1.972 |
| Protein-geometry analysis |  |  |  |
| Ramachandran preferred (%) | 91.8 | 87.9 | 95.5 |
| Ramachandran allowed (%) | 6.9 | 7.6 | 3.9 |
| Ramachandran outliers (%) | 1.3 | 4.5 | 0.6 |
|  |  |  |  |

**Footnotes for Table 1**

*^a,b^*Values in parentheses refer to the highest resolution shell (2.75–2.70 Å) and (2.99–2.94 Å), respectively.

*^c^R*_merge_ = Σ_hkl_Σ_i_ | *I*_i_(*hkl*) – <*I*(*hkl*)> | / Σ_hkl_Σ_i_ *I*_i_(*hkl*)_i_, where *I*(*hkl*) is the intensity of reflection *hkl*, Σ_hkl_ is the sum over all reflections, and Σ_i_ is the sum over i measurements of reflection *hkl*.
